# Dissemination of vancomycin-resistant *Enterococcus faecalis* and *Enterococcus faecium* between humans and fishes

**DOI:** 10.1038/s41598-026-36572-5

**Published:** 2026-03-07

**Authors:** Yasmine H. Tartor, Mohamed Enany, Hassnaa M. Elsheshtawy, Rania M. Kishk, Eman M. Ali, Heba H. Mahboub, Alaaeldin M. Saad, Marwa E. Abo Hashem, Hazem Ramadan

**Affiliations:** 1 Department of Microbiology, Faculty of Veterinary Medicine, Zagazig University, Zagazig, 44511 Egypt; 2 Bacteriology, Immunology and Mycology Department, Faculty of Veterinary Medicine, Suez Canal University, Ismailia, 41522 Egypt; 3 Aquatic Animal Medicine Department, Faculty of Veterinary Medicine, Suez Canal University, Ismailia, Ismailia 41522 Egypt; 4 Microbiology and Immunology Department, Faculty of Medicine, Suez Canal University, Ismailia, 41522 Egypt; 5 Ismalia General Hospital, Ismailia, Egypt; 6 Fish Diseases and Management Department, Faculty of Veterinary Medicine, Zagazig University, Zagazig, 44511 Egypt; 7 Department of Zoonoses, Faculty of Veterinary Medicine, Zagazig University, Zagazig, 44511 Egypt; 8 Hygiene and Zoonoses Department, Faculty of Veterinary Medicine, Mansoura University, Mansoura, 35516 Egypt

**Keywords:** *Enterococcus faecalis*, *Enterococcus faecium*, Vancomycin resistance, Antimicrobial resistance, Virulence genes, Fish, Human, Multilocus sequence typing, Diseases, Microbiology, Molecular biology

## Abstract

Vancomycin-resistant enterococci (VRE) are a major public health concern, yet little is known about their circulation in fish. This study investigated the occurrence, glycopeptide resistance genotypes, virulence characteristics, and sequence types (STs) of VRE isolated from diseased fishes and humans. Isolates were identified using multiplex polymerase chain reaction (PCR) assay and matrix-assisted laser desorption/ionization time-of-flight mass spectrometry (MALDI-TOF MS), and tested for antimicrobial susceptibility. VRE isolates were screened for the presence of glycopeptide resistance genes and eight virulence genes. Multilocus sequence typing (MLST) was determined to assess the clonality of VRE isolates from fishes and humans. Among 60 human samples, 20 *Enterococcus* species isolates (33.33%) including *Enterococcus faecalis* and *Enterococcus faecium* (10 of each), were identified. The overall prevalence of *E. faecalis* was 42.86% (30/70) in *Oreochromis niloticus* and 48.0% (24/50) in *Clarias gariepinus*. *E. faecium* was found in 15.71% (11/70) of *Oreochromis niloticus* and 14.0% (7/50) of *Clarias gariepinus*. Over 50% of human isolates were multidrug resistant (MDR) and 30% exhibited an extensive drug resistant (XDR) phenotype. Fish isolates also displayed high MDR (70.83%) and XDR (29.17%) rates. Forty-nine (53.26%; 34 from fish and 15 from human) isolates were VRE including 30 isolates of *E. faecalis* (VREfs) and 19 isolates of *E. faecium* (VREfm). The *vanA* gene was the most frequent among VREfs (83.33%) and VREfm (100%) isolates. The *vanB* gene was found in 26.67% of VREfs and 15.79% of VREfm. Three out of 10 VREfm (30%) and 2/24 (8.33%) VREfs isolates of fish origin carried both *vanA* and *vanB* genes. *vanC* gene was found in 13.33% (4/30) of VREfs of human and fish origin. One VREfs isolate from human urine carried both *vanA* and *vanC* genes. High frequency of the virulence genes *gelE*, *sprE*, *asa1*, *esp,* and *cylA* were observed; *efa* and *ace* gene was more associated with VREfs, while *hyl* gene was more frequently detected in VREfm. Different combinations of virulence genes suggesting synergistic pathogenic potential. MLST revealed both overlapping and host-specific STs among the examined *Enterococcus* isolates from humans and fish. Experimental infection of *O. niloticus* with VREfs and VREfm caused a 100% and 60% mortality rate within 6 days postinfection, respectively with characteristic disease symptoms. The emergence of VRE and the high prevalence of virulence traits could be regarded as an alarming situation. The call for increased infection control and antibiotic stewardship measures is timely and relevant to combat the spread of VRE in fish and humans.

**Supplementary Information:**

The online version contains supplementary material available at 10.1038/s41598-026-36572-5.

## Introduction

Multidrug-resistant enterococci are one of the superbugs that constitute a worldwide burden and reflect their negative influence on public health^1,2^. Antimicrobial resistance is becoming a progressively public clinical problem for medical workers. Especially, vancomycin-resistant enterococci (VRE) are difficult to control. Enterococci are facultative anaerobic Gram-positive cocci that are commensal organisms inhabit humans and animals’ gastrointestinal tract. Nevertheless, they can induce a diversity of infections^3,4^. Enterococci is a leading cause of several healthcare-associated infections, including central line-linked bloodstream infections, catheter-related urinary tract infections, ventilator-associated pneumonia, and surgical site infections. While most *Enterococcus* isolates are *E. faecalis*, the majority of VRE isolates are *E. faecium*^5^. *Enterococcus* spp. have a substantial ability to transmit and attain resistance genes and virulence factors, stimulate inflammatory processes, and progress resistance to antimicrobial drugs^4^.

VRE infection has been reported to elevate both charge and mortality in comparison to vancomycin-susceptible strains. With cumulative records of vancomycin resistance among *Enterococcus* isolates, upright stewardship joined with violent treatment with targeted antibiotics is essential to cure this regularly encountered infection^6,7^.

Enterococci have been identified as a causative agent of mortalities in the retail fish^8^. *E. faecalis* is a recently occurred fish pathogen distressing numerous cultured fish species, inducing severe congestion and petechial haemorrhages on the internal organs and along the elementary tract^9^. *E. faecalis* have negative impacts on growth, immune response, and intestinal function in various cultured fish species such as silver barb (*Barbonymus gonionotus*), Nile tilapia (*Oreochromis niloticus*), *Barbonymus gonionotus*, and Crucian carp (*Carassius auratus*)^10–13^. *E. faecalis* has been recently isolated from fresh fish and identified its virulence genes^14^. In the fish population, the pathogenicity of enterococci is attributed to the potent adaptive ability which enables bacterium to colonize in the gastrointestinal tract, elevating the chance of gene allocation to the gut microbiota^15^.

Multilocus sequence typing (MLST) has been anticipated as a reproducible scheme to illustrate and recognize bacterial ‘clonal relationships^16^. MLST has been utilized broadly to differentiate genotypes and involving the microbes’ population structure^17,18^ and to categorize potential disease-associated clones or clones linked to antimicrobial resistance traits and virulence factors^19^.

It is worth noting that *Enterococcus* species have become a subject of concern within certain species of fish due to its possible implications for both public health and aquatic ecosystems^20^. Moreover, little is known about the role of the aquatic environment as a reservoir for VRE and the impact of antibiotic use in aquaculture on the emergence of vancomycin resistance. The genetic relatedness of VRE isolates from fishes and humans could enhance the understanding of the findings. This study was designed to answer these questions through (i) determining the frequency of enterococci isolated from the diseased fishes and humans from the same geographical area; (ii) assessing the antibiotic susceptibility profile of the recovered isolates; (iii) identifying virulence genes of VRE isolates; and (iv) investigating the sequence types of VRE isolates from fishes and humans.

## Materials and methods

### Sampling

This study included a total of 60 human samples and 120 fish samples collected during 2024–2025. The Declaration of Helsinki’s guiding principles were closely followed in this study, guaranteeing ethics throughout the research procedure. The protocol of this study was approved by the ethics committee of Suez Canal University, Ismalia, Egypt (SCU-VET-AREC-R-2025034). Moreover, informed consent was obtained from all patients for participation in this study. A total of 60 clinical samples including urine (*n* = 30), wound swabs (*n* = 20), and blood (*n* = 10) were collected from 60 hospitalized patients suffering from urinary tract infection, wounds, and fever^3^. Samples were taken under the guidance of the physician on nephrology ward, surgical ward, and intensive care unit, placed into sterile, labelled containers, and then transported immediately in an icebox to the microbiology laboratory.

Fifty freshwater *Clarias gariepinus* (catfish) and seventy *Oreochromis niloticus* (Nile tilapia) showing signs of septicemia (abdominal distension, diffuse hemorrhages on skin, the operculum, around the mouth, and base of fins, congested protruded anal opening, exophthalmia, eye opacity, as well as dark pigmentation of the skin, detached scales and ulcers)^20^ (at least three fish per tank) were collected from Al Abbasah Farm, Sharkia Governorate. Fresh dead fish were transported in an icebox, while live fish were transported in waterproof bag containing farm water and supplied with oxygen to the bacteriology laboratory for examination. Live fish were humanely euthanized in the laboratory prior to sampling using an overdose (500 mg/L) of sodium bicarbonate–buffered tricaine methanesulfonate (MS-222) (Sigma-Aldrich, St. Louis, MO, USA), in accordance with approved ethical guidelines.

Ismailia city, from which human samples were collected, located in proximity (~ 65 km distance) to the Al Abbasah Farm in Sharkia Governorate.

### Isolation and identification of enterococci

Human and fish samples were cultured on Slanetz and Bartley agar (Oxoid, Basingstoke, UK), incubated at 37 °C for 24 h. A loopful from fish samples (liver, spleen, kidney, and ascitic fluid) were enriched in buffered peptone water for 18 h at 37 °C before plating onto the same selective medium. Pink colonies were picked, subcultured, and subjected to identification based on their morphological and physiological characteristics: Gram staining, bile-esculin, salt tolerance, heat tolerance, catalase, oxidase, motility, and sugar L-arabinose, mannitol, raffinose, sucrose and sorbitol) fermentation tests^21,22^. Further, the isolates were identified using matrix‐assisted laser desorption/ionisation time of flight mass spectrometry (MALDI‐TOF MS, Bruker Daltonics) with MALDI Biotyper software package (version 3.1) and a reference database version 3.1.2.0; 3995 (Bruker Daltonics). According to the manufacturer’s guidelines, an identification log score between 2.00 and 3.00 indicates high-confidence identification, meaning secure genus and species identification with perfect identity. A score in the range of 1.70 to 1.99 suggests secure genus identification but only low-confidence species identification. Scores between 0.00 and 1.69 are considered unreliable and indicate ambiguous identification.

The identity of the presumptive *E. faecalis* and *E. faecium* isolates was further confirmed by multiplex polymerase chain reaction (PCR) assay using the species-specific primers (Table S1) targeting *ddl* (D-ala-nine-D-alanine ligase) genes^23^.

### Antimicrobial susceptibility testing of *E. faecalis *and *E. faecium* isolates

Antimicrobial susceptibility testing was performed using disc diffusion method following the criteria provided by the Clinical and Laboratory Standards Institute (CLSI)^24^. The following antibiotic discs (Oxoid, Ltd., Basingstoke, England) for the most frequently used and prescribed antibiotics were selected for testing and identification of multidrug-resistant (MDR), extensively drug-resistant (XDR), or pandrug-resistant (PDR) *Enterococcus* species according to Magiorakos et al.^25^: linezolid (LNZ, 5 µg), tigecycline (TGC, 10 µg), teicoplanin (TIC, 30 µg), vancomycin (VAN, 30 µg), gentamicin (CN, 120 µg), streptomycin (STP, 300 µg), daptomycin (DPT, 30 µg), imepinem (IMP, 10 µg), meropenem (MRP,10 µg), ciprofloxacin (CIP, 5 µg), levofloxacin (LEV, 5 µg), nitrofurantoin (F, 20 µg), amoxicillin (AMX, 10 µg), ampicillin (AMP, 10 µg), tetracycline (TE, 30 µg), clindamycin (CLN, 20 µg), erythromycin (ERT, 15 µg), quinupristin/dalfopristin (QD, 15 μg), chloramphenicol (CHL, 20 µg), azithromycin (AZT, 15 µg), ofloxacin (OFX, 5 µg), and norfloxacin (NOR, 10 µg). The minimum inhibitory concentration (MIC) values of VAN and TIC (Sigma, MO, USA) were determined by the broth microdilution method according to the CLSI recommendations^24^. The isolates were considered VAN resistant when MIC was ≥ 32, intermediate at 8–16, and VAN sensitive at ≤ 4 μg/ml. The breakpoints for TIC were ≤ 8 (sensitive), 16 (intermediate), and ≥ 32 (resistant). For the disk diffusion method, the control strain is *Staphylococcus aureus* ATCC 25923 (American Type Culture Collection, Manassas, VA, USA) and for MIC testing, *E. faecalis* ATCC 29212 was used. Multiple antibiotic resistance (MAR) index of each isolate was determined as previously described^26^.

### Detection of vancomycin resistance genes

The identification of the genes responsible for vancomycin resistance (Table S1) was investigated using a multiplex PCR assay^23,27^. After overnight culture of each isolate in brain heart infusion broth (BBL, Difco, BD, Sparks, MD), cells were harvested (15,000 × g, 5 min), and DNA was extracted using QIAamp DNA Mini Kit (QIAGEN GmbH, Hilden, Germany) according to the manufacturer’s instructions. DNA template (6 μL) was added to 25 μL Emerald Amp GT PCR mastermix (Takara, Japan), 1 μL (20 pmol) of each forward and reverse primers (Invitrogen, Carlsbad, CA, USA), and 13 μL of molecular grade water. Amplification was performed in a T3 Thermal cycler (Biometra GmbH, Goettingen, Germany) using the following conditions: 94 °C for 3 min; 30 cycles of 94 °C for 1 min, 54 °C for 1 min, and 72 °C for 1 min; and a final extension at 72 °C for 7 min. Vancomycin-resistant reference strains (*E. faecium* ATCC 51559 and *E. faecalis* ATCC 51299) and the reaction mixture without DNA template were used as positive and negative controls, respectively. Amplified products were analyzed by electrophoresis on 1% agarose gel containing 0.5 µg/mL ethidium bromide and were visualized using gel documentation system (BioRad, UK).

### Screening for virulence genes in vancomycin-resistant enterococci

VAN-resistant isolates were screened for the presence of virulence genes including *gelE, sprE, esp, efaA, ace, asaI, hyl,* and *cylA*. Multiplex PCR assay as indicated by Yang et al.^28^ was performed for detection of *esp*, *gelE*, *asa1*, *hyl*, and *cylA* genes. PCR conditions for amplification reactions were initial denaturation at 95 °C for 3 min; 30 cycles at 94 °C for 1 min, 54 °C for 1 min (58 °C for *efa*A and *ace*; 56 for *sprE* (Table S1)) , and 72 °C for 1 min; and extension at 72 °C for 10 min. PCR products were analyzed by electrophoresis in 1.2% agarose gel. Then BioRad gel documentation system was used for the documentation.

### Multilocus sequence typing

The VAN-resistant isolates with high MAR index were further characterized by amplification and sequencing of seven housekeeping genes of *E. faecalis* and *E. faecium* (Table S1) as previously described^29,30^. The obtained sequences were quality-trimmed and assembled into consensus sequences for each locus. These sequences were then compared to the respective PubMLST database (*E. faecalis*, https://pubmlst.org/organisms/enterococcus-faecalis; *E. faecium,* https://pubmlst.org/organisms/enterococcus-faecium) to assign allele numbers. The combination of allelic numbers given for the unique sequences was used to identify the sequence type (ST) of each isolate using the multilocus sequence typing (MLST) scheme of *E. faecalis* and *E. faecium*.

### Pathogenicity test

Experimental infection of *O. niloticus* (50 ± 2.5 g; Fish Research Unit, Faculty of Veterinary Medicine, Zagazig University, Egypt) using virulent strains of VREfs (group 1) and VREfm (group 2) was done as previously described^31^. All procedures were conducted in accordance with the United Kingdom Animal (Scientific Procedures) Act 1986 in compliance with the ARRIVE guidelines and approved by institutional ethical review committees (SCU-VET-AREC-R-2025034). Three groups of fish were kept in three 60-L glass aquaria (10 fish/aquaria) with well aerated flowing water. Fish were fed with commercial pellets and acclimated for 2 weeks before experimental infection with daily monitoring of dissolved oxygen, temperature, and pH of water. Bacterial suspension (2.1** × **10^8^) was prepared in sterile physiological saline and then injected intraperitonially into two groups while group 3 served as a control and injected with saline. The injected fish were observed for 14 days, and the mortality rate was recorded. Infection was confirmed by re-isolation of bacteria from the internal organs (kidney, liver, and spleen) of dead fish using Slanetz and Bartley agar medium followed by biochemical identification of isolates.

### Data analysis

The data were analyzed using SPSS version 26 (IBM Corp, Armonk, NY, USA). The Chi-square (*χ*^2^) test was used to analyze the variations in the antimicrobial resistance patterns of the recovered isolates from human and fishes. This test was also used to compare the frequencies of vancomycin resistance and virulence genes among the isolated *Enterococcus* from humans and fishes. A heatmap was constructed to visualize the distribution of antimicrobial resistance phenotypes and virulence genes across *E. faecalis* and *E. faecium* isolates. The data were converted to a binary format, where 1 indicates antimicrobial resistance or the presence of a virulence gene, and 0 indicates antimicrobial susceptibility or its absence. Binary data were imported into R-software version 4.4.3 (https://www.r-project.org/) and heatmap was generated using the “pheatmap” and “RColorBrewer” package^32^. A Kaplan Meier survival analysis in GraphPad Prism (version 9.0, GraphPad Software Inc., La Jolla, CA, United States) was used to analyze the survival rate. Statistical significance was set at *p* value less than 0.05.

## Results

### Prevalence of enterococci from humans and fishes samples

Twenty isolates of enterococci were recovered from 60 human clinical samples with an overall prevalence rate of 33.33%. Four *E. faecium* isolates were obtained from blood samples (40%) and five *E. faecium* isolates (25%) from wound samples. Eleven isolates were obtained from urine samples (36.7%) including 10 *E. faecalis* and one *E. faecium* isolates.

Table 1 lists the prevalence of *E. faecalis* and *E. faecium* isolates in the 480 fish samples examined from 120 fish. We recovered 143 *E. faecalis* and 42 *E. faecium* isolates (1–4 isolates per fish) including 74 *E. faecalis* isolates (derived from 30/70 *O. niloticus*); 26 *E. faecium* isolates (from 11/70 fish) and 69 *E. faecalis* isolates (derived from 24/50 *Clarias gariepinus*) and 16 *E. faecium* isolates from 7/50 fish. The overall prevalence of *E. faecalis* was 42.86% in *O. niloticus* and 48.0% in *C. gariepinus*. *E. faecium* was found in 15.71% of *O. niloticus* and 14.0% of *C. gariepinus* examined.

**Table 1:** Frequency of *Enterococci* in *Oreochromis niloticus* and *Clarias gariepinus* samples.

Organ	*Oreochromis niloticus*				*Clarias gariepinus*			
	No. of samples	No. (%) of positive samples for enterococci	No. of *E. faecalis* recovered	No. of *E. faecium* recovered	No of samples	No (%) of positive samples for enterococci	No. of *E. faecalis* recovered	No. of *E. faecium* recovered
Liver	70	21 (30%)	16	5	50	31 (62%)	24	7
Kidney	70	26 (37.14%)	20	6	50	23 (46%)	21	2
Spleen	70	25 (35.71%)	19	6	50	26 (52%)	19	7
Ascitic fluid	70	28 (40%)	19	9	50	5 (10%)	5	0
Total	280	100 (35.71%)	74	26	200	85 (42.5%)	69	16

All isolates produced pink colonies on Slanetz and Bartley agar, Gram-positive cocci, grew in the presence of 6.5% NaCl, 40% bile salts, hydrolyses the aesculin, tolerates the temperature of 60 °C, non-motile, mannitol fermentative, *E. faecalis* ferment sorbitol, *E. faecium* ferment arabinose, catalase and oxidase negative. Using MALDI-TOF MS, all isolates (100%) were identified as *Enterococcus* spp. Of these, 20 human isolates and 67 fish isolates (94.57%) were identified to the species level with log scores between 2.00 and 3.00. The remaining five isolates (5.43%) were identified only at the genus level, with log scores ranging from 1.80 to 1.93.

### Antibiogram profiling of humans and fishes enterococci isolates

**Fig. 1:**
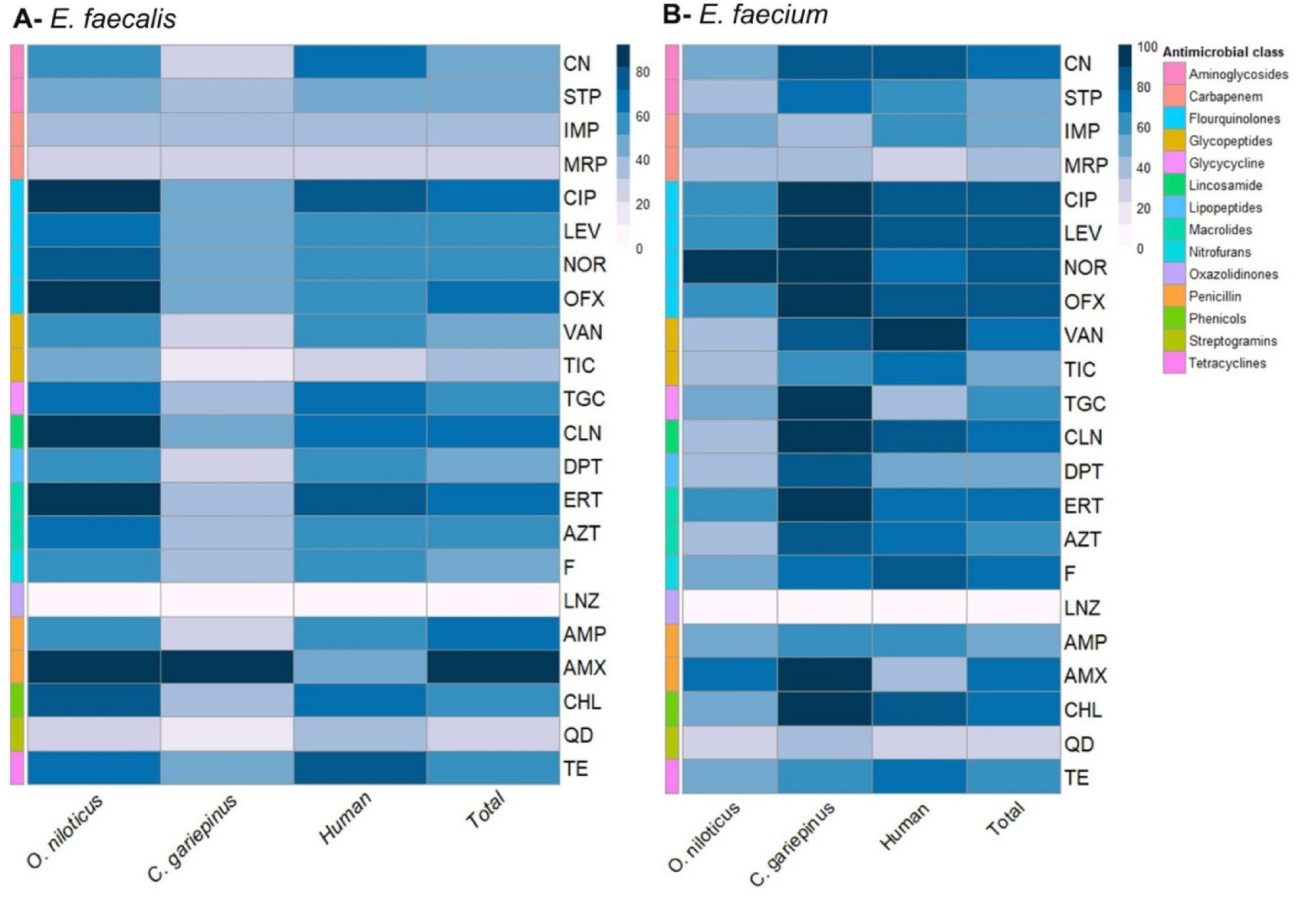
Heatmap showing the antimicrobial resistance profile of *E. faecalis* (**A**), and *E. faecium* (**B**) isolates from *Oreochromis niloticus, Clarias gariepinus*, and humans. The scale on the right of each heatmap refers to the frequency of resistance to each antibiotic. The frequency increased with the increased intensity of colour. AMP: AMP: Ampicillin, AMX: Amoxicillin, CIP: Ciprofloxacin, LEV: Levofloxacin, NOR: Norofloxacin, OFX: Ofloxacin, LNZ: Linezolid, TGC: Tigecycline, CN: Gentamicin, STP: Streptomycin, IMP: Imipenem, MRP: Meropenem, ERT: Erythromycin, AZT: Azithromycin, CLN: Clindamycin, VAN: Vancomycin, TIC: Teicoplanin, CHL: Chloramphenicol, F: Nitrofurantoin, TE: Tetracycline, DPT: Daptomycin, QD: Quinupristin-dalfopristin.

The antibiogram profiling of 20 human isolates, 30 *E. faecalis* and 11 *E. faecium* isolates from *O. niloticus* (one isolate per fish), and 24 *E. faecalis* and 7 *E. faecium* isolates from *C. gariepinus* were determined against 22 antibiotics (Table 2 and Fig. 1A and B). Human and fish isolates were susceptible to linezolid (100%) and quinupristin-dalfopristin (65% and 75%, respectively). Human isolates were highly resistant to ciprofloxacin (80%), gentamicin, erythromycin, clindamycin, vancomycin, chloramphenicol, and tetracycline (75% each), levofloxacin, ofloxacin, and nitrofurantoin (70% each), norofloxacin (65%), and ampicillin (60%). Over 50% of human isolates were resistant to at least one antibiotic from at least three different classes of antimicrobial agents and considered as MDR with MAR index ranged from 0.27 to 0.86. Six isolates (30%) exhibited an XDR phenotype as being unsusceptible to at least one antibiotic of all tested antimicrobial classes but remained susceptible to one class (oxazolidinones).

**Table 2:** Antimicrobial resistance profile of *E. faecalis* and *E. faecium* isolates from humans and fishes.

Antibiotics	No. of resistant isolates (%)											
	*Oreochromis niloticus (n = 41)				*Clarias gariepinus (n = 31)				Human (n = 20)			
	*E. faecalis* (n = 30)	*E. faecium* (n = 11)	*p* value	Total (n = 41)	*E. faecalis* (n = 24)	*E. faecium* (n = 7)	*p* value	Total (n = 31)	*E. faecalis* (n = 10)	*E. faecium* (n = 10)	*p* value	Total (n = 20)
Ampicillin	17 (56.67)	5 (45.45)	0.725	22 (53.7)	7 (29.17)	4 (57.14)	0.21	11 (35.5)	6 (60)	6 (60)	1	12 (60)
Amoxicillin	27 (90)	8 (72.73)	0.316	35 (85.4)	22 (91.67)	7 (100)	1	29 (93.5)	5 (50)	4 (40)	1	9 (45)
Ciprofloxacin	26 (86.67)	7 (63.63)	0.178	33 (80.5)	11 (45.83)	7 (100)	0.025*	18 (58.1)	8 (80)	8 (80)	1	16 (80)
Levofloxacin	20 (66.67)	7 (63.63)	1	27 (65.9)	11 (45.83)	7 (100)	0.025*	18 (58.1)	6 (60)	8 (80)	0.628	14 (70)
Norofloxacin	23 (76.67)	10 (90.91)	0.412	33 (80.5)	10 (41.67)	7 (100)	0.009**	17 (54.8)	6 (60)	7 (70)	1	13 (65)
Ofloxacin	26 (86.67)	7 (63.63)	0.178	33 (80.5)	11 (45.83)	7 (100)	0.025*	18 (58.1)	6 (60)	8 (80)	0.628	14 (70)
Linezolid	0	0	NA	0	0	0	NA	0	0	0	NA	0
Tigecycline	19 (63.33)	5 (45.45)	0.476	24 (58.5)	9 (37.5)	7 (100)	0.007**	16 (51.6)	7 (70)	4 (40)	0.37	11 (55)
Gentamicin	17 (56.67)	5 (45.45)	0.725	22 (53.7)	6 (25.00)	6 (85.71)	0.007**	12 (38.71)	7 (70)	8 (80)	1	15 (75)
Streptomycin	14 (46.67)	4 (36.36)	0.726	18 (43.9)	8 (33.33)	5 (71.43)	0.099	13 (41.9)	5 (50)	6 (60)	1	11 (55)
Imipenem	10 (33.33)	5 (45.45)	0.716	15 (36.6)	8 (33.33)	3 (42.86)	0.676	11 (35.5)	4 (40)	6 (60)	0.656	10 (50)
Meropenem	7 (23.33)	4 (36.36)	0.445	11 (26.8)	6 (25.00)	3 (42.86)	0.639	9 (29)	3 (30)	3 (30)	1	6 (30)
Erythromycin	26 (86.67)	7 (63.63)	0.178	33 (80.5)	9 (37.5)	7 (100)	0.007**	16 (51.6)	8 (80)	7 (70)	1	15 (75)
Azithromycin	21 (70.00)	4 (36.36)	0.074	25 (60.9)	8 (33.33)	6 (85.71)	0.028*	14 (45.2)	6 (60)	7 (70)	1	13 (65)
Clindamycin	26 (86.67)	4 (36.36)	0.003**	30 (73.2)	10 (41.67)	7 (100)	0.083	17 (54.8)	7 (70)	8 (80)	1	15 (75)
Vancomycin	17 (56.67)	4 (36.36)	0.306	21 (51.22)	7 (29.17)	6 (85.71)	0.012*	13 (41.9)	6 (60)	9 (90)	0.303	15 (75)
Teicoplanin	15 (50)	4 (36.36)	0.499	19 (46.3)	4 (16.67)	4 (57.14)	0.053	8 (25.8)	3 (30)	7 (70)	0.179	10 (50)
Chloramphenicol	22 (73.33)	6 (54.55)	0.28	28 (68.3)	9 (37.5)	7 (100)	0.007**	16 (51.6)	7 (70)	8 (80)	1	15 (75)
Nitrofurantoin	17 (56.67)	6 (54.55)	1	23 (56.1)	8 (33.33)	5 (71.43)	0.099	13 (41.9)	6 (60)	8 (80)	0.628	14 (70)
Tetracycline	19 (63.33)	5 (45.45)	0.476	24 (58.5)	11 (45.83)	4 (57.14)	0.685	15 (48.4)	8 (80)	7 (70)	1	15 (75)
Daptomycin	17 (56.67)	4 (36.36)	0.306	21 (51.2)	6 (25)	6 (85.71)	0.007**	12 (38.7)	6 (60)	5 (50)	1	11 (55)
Quinupristin-dalfopristin	8 (26.67)	3 (27.27)	1	11 (26.8)	4 (16.67)	3 (42.86)	0.302	7 (22.6)	4 (40)	3 (30)	1	7 (35)

NA: non-applicable.

*p < 0.05, **p < 0.01, ***p < 0.001. *One isolate per fish was tested.

As revealed in Table 2, *E. faecium* isolates from human samples showed higher resistance rates to all tested antibiotics than *E. faecalis* isolates except amoxicillin, tigecycline, erythromycin, daptomycin, tetracycline, and quinupristin-dalfopristin. There were no statistically significant differences (*p* > 0.05) in the antimicrobial resistance patterns of enterococcal isolates recovered from human samples to all tested antibiotics (Table 2).

All fish isolates were highly resistant to amoxicillin (88.89%), ciprofloxacin, ofloxacin (70.83%, each), norofloxacin (69.44%), erythromycin (68.05%), levofloxacin (62.5%), clindamycin (65.28%), and chloramphenicol (61.11%). Moreover, over 50% of the isolates were resistant to tigecycline (55.56%), azithromycin and tetracycline (54.17% each), and nitrofurantoin (50%). Notably, of the 72 tested fish isolates, 21 (29.17%) were XDR with MAR index ranged from 0.86 to 0.95 and 51 isolates (70.83%) were MDR with MAR index ranged from 0.14 to 0.86. The isolates were sensitive to meropenem (72.22%), streptomycin (56.94%), imipenem (63.89%), ampicillin (54.17%), gentamicin (52.78%), daptomycin (54.17%), teicoplanin (62.5%), and vancomycin (52.77%).

Overall, higher resistance rates to the tested antibiotics were found in *E. faecium* isolates from *C. gariepinus* than in *E. faecalis* isolates (Table 2). However, *E. faecalis* isolates from *O. niloticus* showed higher resistance rates to all tested antibiotics than *E. faecium* isolates except norofloxacin (76.67% vs. 90.91%), imipenem (33.33% vs. 45.45%), meropenem (23.33% vs. 36.36%), quinupristin-dalfopristin (26.67% vs. 27.27%). Statistical analysis revealed significant variations in the antimicrobial resistance patterns of enterococcal isolates recovered from *O. niloticus* samples to clindamycin (*p* = 0.003) (Table 2). Moreover, there were statistically significant differences (*p* < 0.05) in the antimicrobial resistance patterns of enterococcal isolates recovered from *C. gariepinus* to ciprofloxacin, levofloxacin, norofloxacin, ofloxacin, tigecycline, gentamicin, erythromycin, azithromycin, vancomycin, chloramphenicol, and daptomycin antibiotics (Table 2).

Statistical analysis revealed significant variations (*p* < 0.05) in the antimicrobial resistance patterns of *E. faecalis* isolates recovered from *O. niloticus, C. gariepinus*, and human samples to amoxicillin, ciprofloxacin, norofloxacin, ofloxacin, gentamicin, erythromycin, azithromycin, clindamycin, teicoplanin, chloramphenicol, daptomycin antimicrobials (Table S2). Similarly, there were significant variations (*p* < 0.05) in the antimicrobial resistance patterns of *E. faecium* isolates recovered from *O. niloticus, C. gariepinus*, and human samples to amoxicillin, tigecycline, clindamycin, and vancomycin antimicrobials (Table S2).

### Prevalence of vancomycin-resistant *E. faecalis *and* E. faecium* in humans and fishes

**Fig. 2:**
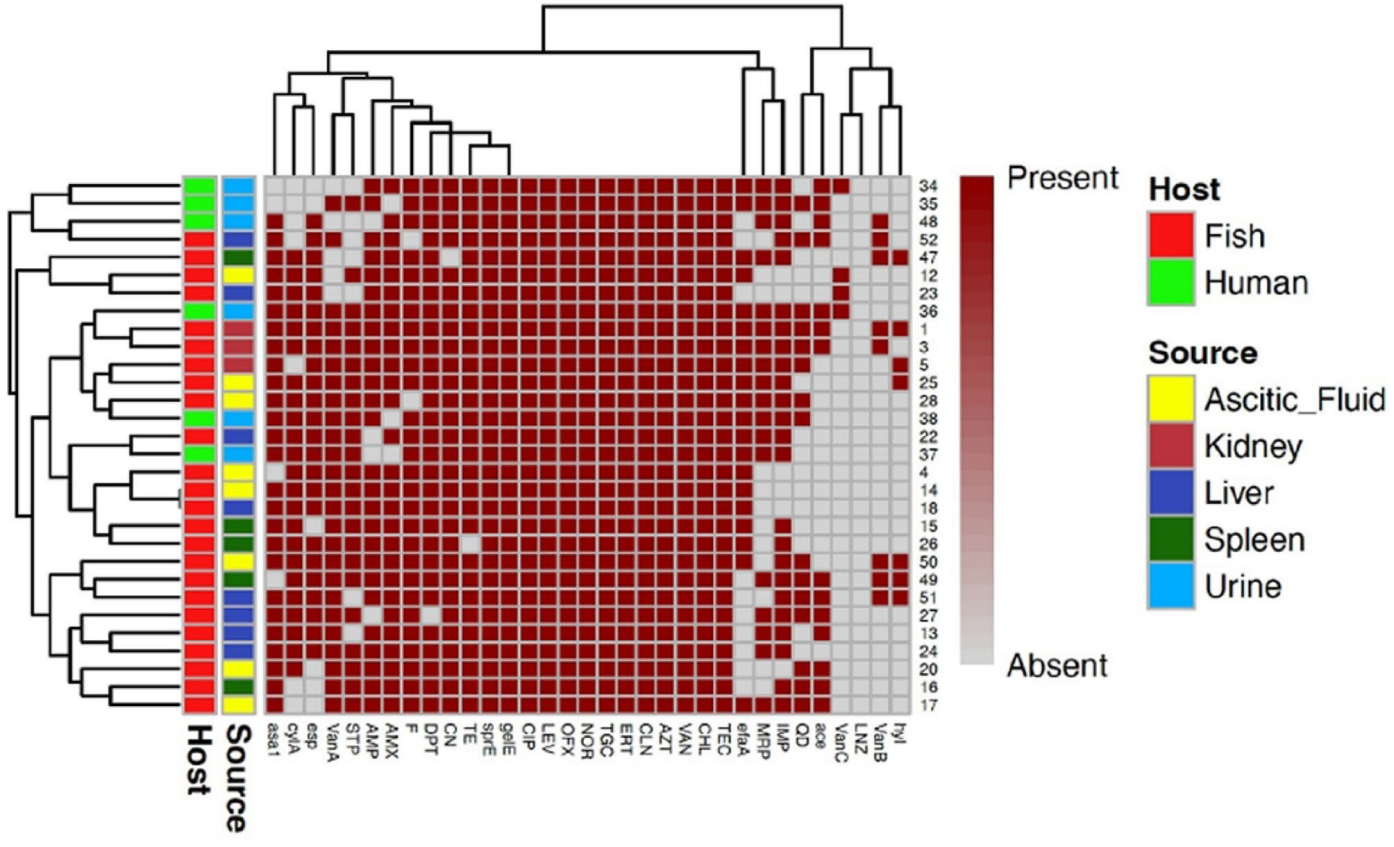
A heatmap supported by a dendrogram for vancomycin resistant *E. faecalis* (*n* = 30) from humans and fishes showing their antimicrobial resistance phenotypes and the distribution of virulence genes. AMP: Ampicillin, AMX: Amoxicillin, CIP: Ciprofloxacin, LEV: Levofloxacin, NOR: Norofloxacin, OFX: Ofloxacin, LNZ: Linezolid, TGC: Tigecycline, CN: Gentamicin, STP: Streptomycin, IMP: Imipenem, MRP: Meropenem, ERT: Erythromycin, AZT: Azithromycin, CLN: Clindamycin, VAN: Vancomycin, TIC: Teicoplanin, CHL: Chloramphenicol, F: Nitrofurantoin, TE: Tetracycline, DPT: Daptomycin, QD: Quinupristin-dalfopristin.

A total of 49 vancomycin-resistant enterococci (VRE) isolates were detected including 34 (47.22%) enterococci from fish samples and 15 (75%) isolates from humans (Figs. 1 and 2). Twenty-seven vancomycin-resistant fish isolates (79.41%) were classified as high-level vancomycin-resistant (HLVR) with MICs ≥ 256 μg/mL, while seven isolates showed MICs ≥ 32 μg/mL. Four (26.67%) vancomycin-resistant isolates from human samples showed MICs ≥ 32 μg/mL and 73.33% of isolates displayed MIC ≥ 256 μg/mL. The teicoplanin MICs ranged from 16 to 46 μg/mL.

Of 34 VRE recovered from fish samples, 24 (70.59%) isolates were identified as *E. faecalis* (VREfs) and 10 isolates (29.41%) were *E. faecium* (VREfm). However, 60% (9/15) of VRE from human samples were *E. faecium* and 40% (6/15) were *E. faecalis*.

The *vanA* gene was the most frequent among VREfs and VREfm isolates (Table 3). It was detected in 83.33% (25/30) of VREfs and 100% (19/19) of VREfm. The *vanB* gene was found in 26.67% (8/30) of VREfs and 15.79% (3/19) of VREfm. Three out of 10 VREfm (30%) and 2/24 (8.33%) VREfs isolates of fish origin carried both *vanA* and *vanB* genes. Notably, the *vanC* gene was found in 13.33% (4/30) of VREfs of human and fish origin (2 from each) but not detected in VREfm. One VREfs isolate from human urine carried both *vanA* and *vanC* genes. Among 21 VRE isolates, 12 (57.1%) isolates were positive for the presence of van genes. Ten *E. faecium* (47.6%) isolates carried *vanA* and 2 (9.5%) *Enterococcus* species carried *vanC*.

**Table 3:** Frequency of vancomycin resistance genes and virulence genes in *Enterococcus* species isolated from humans and fishes.

Genes	No. (%) of *E. faecalis*				No. (%) of *E. faecium*				^§^*p* value
	*O. niloticus* (*n* = 17)	*C. gariepinus* (*n* = 7)	Human (*n* = 6)	Total (*n* = 30)	*O. niloticus* (*n* = 4)	*C. gariepinus* (*n* = 6)	Human (*n* = 9)	Total (*n* = 19)	
*Van A*	16	5	4	25 (83.33)	4	6	9	19 (100.00)	0.06
*Van B*	5	2	1	8 (26.67)	2	1	0	3 (15.79)	0.37
*Van C*	1	1	2	4 (13.33)	0	0	0	0 (0.00)	0.09
*gelE*	17	7	6	30 (100.00)	2	5	7	14 (73.68)	0.003*
*efa*	11	4	5	20 (66.67)	0	1	3	4 (21.05)	0.001*
*asa1*	16	6	4	26 (86.67)	2	4	8	14 (73.68)	0.25
*esp*	14	6	4	24 (80.00)	2	5	7	14 (73.68)	0.6
*sprE*	17	7	6	30 (100.00)	2	5	7	14 (73.68)	0.003*
*hyl*	4	3	0	7 (23.33)	2	3	1	6 (31.58)	0.52
*cylA*	15	5	3	23 (76.67)	4	6	7	17 (89.47)	0.26
*ace*	7	3	4	14 (46.67)	1	1	2	4 (21.05)	0.07

^**§**^The statistical significance of the data was determined using Chi*-*square test, *p value < 0.05 was considered significant.

**Fig. 3:**
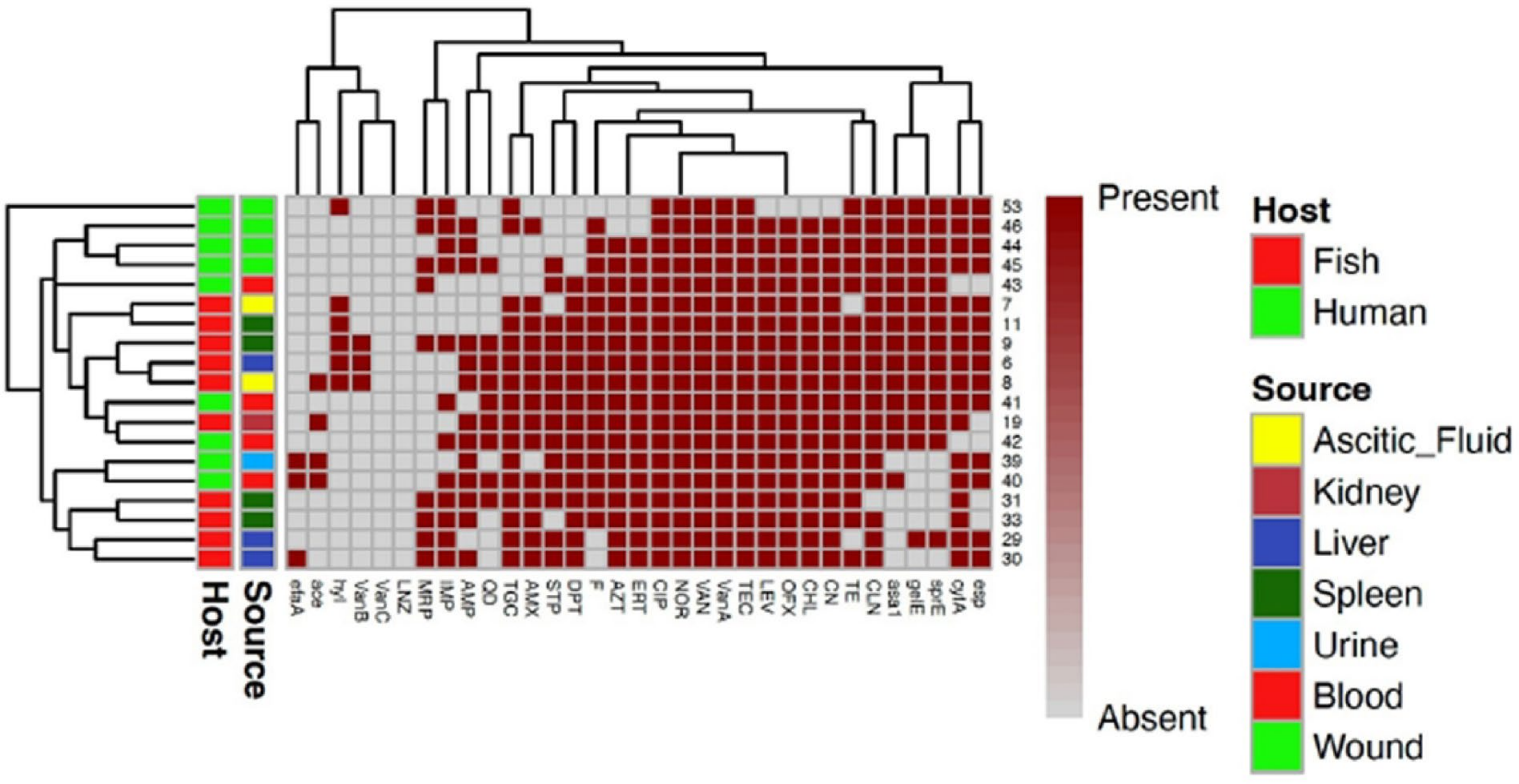
A heatmap supported by a dendrogram for vancomycin resistant *E. faecium* (*n* = 19) from humans and fishes showing their antimicrobial resistance phenotypes and the distribution of virulence genes. AMP: Ampicillin, AMX: Amoxicillin, CIP: Ciprofloxacin, LEV: Levofloxacin, NOR: Norofloxacin, OFX: Ofloxacin, LNZ: Linezolid, TGC: Tigecycline, CN: Gentamicin, STP: Streptomycin, IMP: Imipenem, MRP: Meropenem, ERT: Erythromycin, AZT: Azithromycin, CLN: Clindamycin, VAN: Vancomycin, TIC: Teicoplanin, CHL: Chloramphenicol, F: Nitrofurantoin, TE: Tetracycline, DPT: Daptomycin, QD: Quinupristin-dalfopristin.

Most VRE isolates from fish presented XDR phenotype, 61.76% (21/34), and 38.24% were MDR (13/34) while 60% (9/15) of VRE from humans presented MDR phenotype and 40% (6/15) presented XDR phenotype. Resistance to quinupristin/dalfopristin was detected in 50% (15/30) and 47.37% (9/19) of the VREfs and VREfm isolates, respectively. No resistance to linezolid was observed (Figs. 2 and 3).

### Distribution of virulence genes among vancomycin-resistant enterococci isolates

VREfs and VREfm isolates exhibiting a different preference of the virulence genes, including aggregation substance (*asa1*), surface protein (*espE*), collagen-binding protein (*ace*), serine protease (*sprE*), endocarditis antigen (*efaA*), cytolysin activator (*cylA*), gelatinase (*gelE*), and hemolysin (*hyl*) (Table 3). High frequency of the virulence genes *gelE*, *sprE*, *asa1*, *esp,* and *cylA* were observed; *efa* and *ace* gene was more associated with VREfs, while *hyl* gene was more frequently detected in VREfm. The most frequent virulence genes in VREfs were *gelE* and *sprE* (100.0%), followed by *asa1* (86.67%), *esp* (80.00%) and *cylA* (76.67%). However, *cylA* gene was the most frequent gene detected in VREfm isolates (89.47%) followed by *gelE*, *sprE, asa1*, and *esp* (73.68%). As shown in Table 3, the frequency of *gelE*, *efa*, and *sprE* genes among *E. faecalis* isolates were significantly higher than VREfm (*P* < 0.05).

Several different combinations of virulence genes were found in VREfs and VREfm (Figs. 2 and 3). The most frequent combination in VREfs was *gelE-sprE-asa1-cylA-efaA-esp* (8/30, 26.67%), and *gelE-sprE-asa1-cylA-esp-hyl* in VREfm (5/19, 26.32%). In other words, all isolates harbored at least two of the screened genes except two VREfm isolates, with the majority harboring between five and seven genes, while one isolate harbored all eight screened genes (Table 4).

**Table 4:** Virulence genes profile in *E. faecalis* and *E. faecium* isolates from humans and fishes.

Virulence genes pattern	Total isolates	Human isolates showing the pattern		Fish isolates showing the pattern	
		No. of *E. faecalis*	No. of *E. faecium*	No. of *E. faecalis*	No. of *E. faecium*
*gelE-sprE-ace-asa1-cylA-efaA-esp-hyl*	1			1	
*gelE-sprE-asa1-cylA-efaA-esp-hyl*	3			3	
*gelE-sprE-ace-asa1-cylA-efaA-esp*	2	1		1	
*gelE-sprE-ace-asa1-cylA-esp-hyl*	2			1	1
*gelE-sprE-asa1-cylA-efaA-esp*	8	2		6	
*gelE-sprE-asa1-efaA-esp-hyl*	1			1	
*gelE-sprE-asa1-cylA-esp-hyl*	5		1		4
*gelE-sprE-ace-asa1-cylA-esp*	2			2	
*gelE-sprE-ace-cylA-esp-hyl*	1			1	
*gelE-sprE-asa1-cylA-efaA*	1			1	
*gelE-sprE-cylA-efaA-esp*	1			1	
*gelE-sprE-ace-asa1-efaA*	1			1	
*gelE-sprE-ace-asa1-cylA*	2			1	1
*gelE-sprE-asa1-cylA-esp*	6		4	2	
*gelE-sprE-ace-asa1-esp*	2	1		1	
*ace-asa1-cylA-efaA-esp*	1		1		
*gelE-sprE-cylA-esp*	1				1
*gelE-sprE-ace-asa1*	1			1	
*gelE-sprE-ace-efaA*	2	2			
*ace-cylA-efaA-esp*	1		1		
*gelE-sprE-asa1*	2		2		
*cylA-efaA-esp*	1				1
*cylA*	2				2

#### Molecular typing

MLST results demonstrated the presence of various STs among the ten examined isolates from humans and fishes. Of the four STs identified for *E. faecalis*, ST21 was found in both human and fish isolates. The other STs were identified in isolates sourced from a single host: ST273 was detected in fish, and ST283 and ST878 were found in human isolates. For *E. faecium*, two different STs existed: ST218 from a human isolate and ST583 from a fish isolate (Table S3).

### Pathogenicity of VAN-resistant enterococci in *Oreochromis niloticus*

**Fig. 4:**
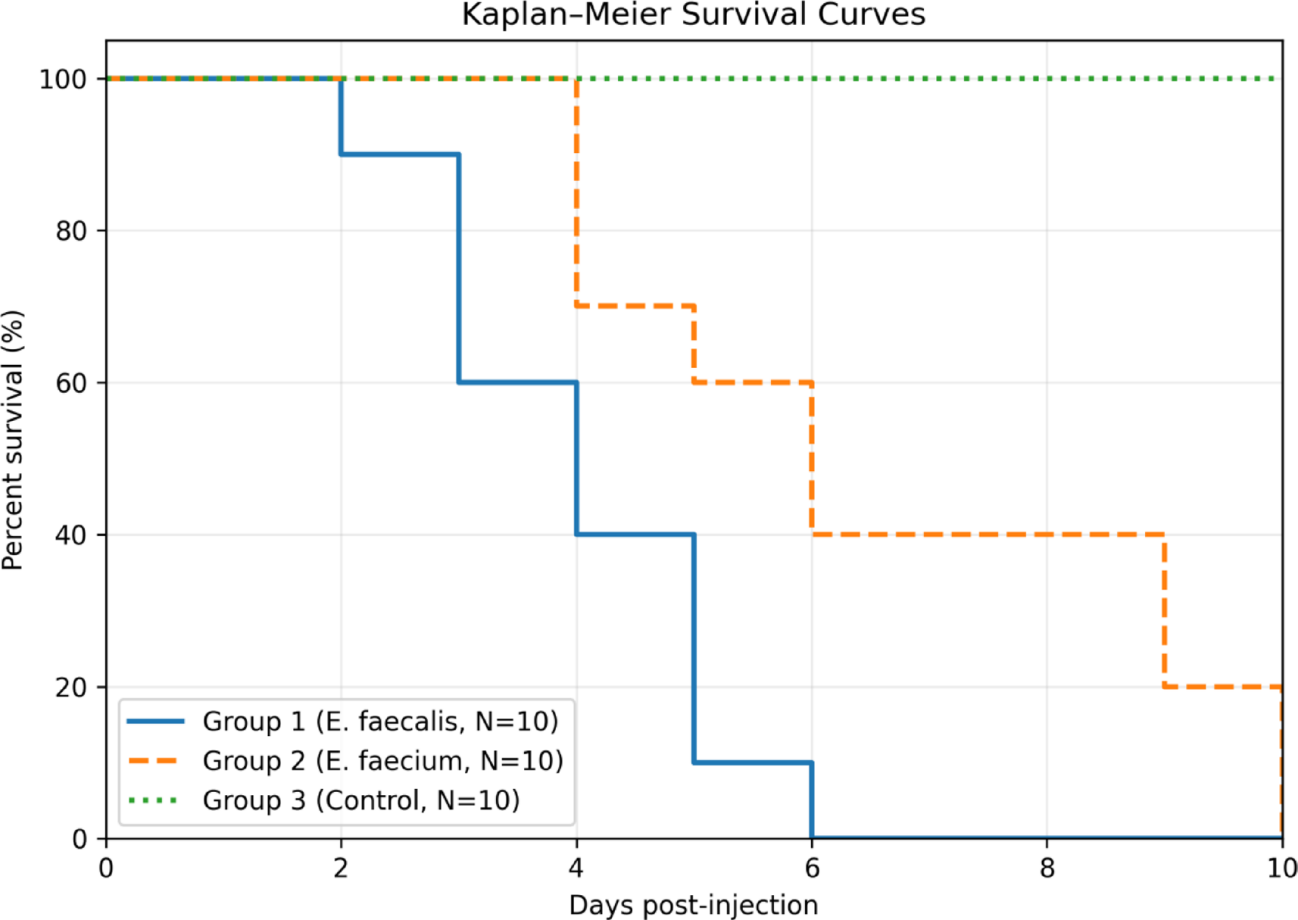
Survival rate of *Oreochromis niloticus* experimentally infected with virulent strains of vancomycin resistant *E. faecalis* (group 1) and *E. faecium* (group 2) are shown. Group 3 served as the negative control and was inoculated with sterile saline.

*Oreochromis niloticus* were experimentally infected with VREfs and VREfm isolates possessing gelatinase (*gelE*), cytolysin (*cylA*), enterococcal surface protein (*esp*), hyaluronidase (*hyl*) and aggregation (*asa1*) genes. The clinical signs and postmortem lesions were like those observed in naturally infected fish. The fish showed darkening of the body, hemorrhage on body and fins, opacity of eye, exophthalmia, enlargement and congestion of liver, congested kidney, and distended gall bladder (Fig. S1). There is a significant difference (*p* < 0.01) in the mortality pattern of group 1 (VREfs group) and 2 (VREfm group). VREfs induced the highest mortality (100%) followed by VREfm (60%) within 6 days of infection (Fig. 4). Mortality in VREfs group started on the 2^nd^day post infection and continued until day 6 post infection. The highest mortality rate was found in the 3^rd^ day post infection in VREfs group. While mortality started on day 4 post infection in VREfm group (Group 3) and continued up to day 10 post infection (Fig. 4). No mortalities or abnormal clinical signs were observed on fish in the control negative group (Group 3).

## Discussion

Approximately 60 of human infections originate from animals. Among these *Enterococcus* species, especially MDR and virulent strains pose a significant concern. These difficult-to-treat bacteria have even been detected in healthy wild animals, which can serve as reservoirs and potentially transmit them to humans^33^. *E. faecalis* and *E. faecium* are the primary culprits behind most human enterococcal infections, especially hospital-acquired ones, and these are often highly resistant to many antibiotics^34^. This study investigated the frequency, resistance phenotypes, *van* genes, MLST, and virulence factors of *E. faecalis* and *E. faecium* in humans and fishes. Our findings revealed the occurrence of *Enterococcus* species in 33.3% of human clinical samples. *E. faecalis* was equally proportion to *E. faecium* (16.66% each). Similarly, Ayobami et al.^35^ recorded the rise of *E. faecalis* clinical isolates in several European countries to 17.3%. However, Mubarak et al.^14^ found that the prevalence of *E. faecalis* and *E. faecium* in diarrheal patients in Egypt were 23.23% and 10.83%, respectively. A higher percentage of *E. faecium* (56.9%) and *E. faecalis* (43.1%) were isolated from hospitalized patients in China^36^. Moreover, Uda et al.^37^ detected *E. faecium* (51%) and *E. faecalis* (47%) in patients infected with enterococcal bacteremia. The incidence of *Enterococcus* infections varied according to the study location, population, and the associated risk factors^38^.

In our study, *Enterococcus* spp. were detected in 60% of the 120 fish examined, based on 480 samples collected from liver, kidney, spleen, and ascitic fluid. *E. faecalis* was more prevalent than *E. faecium* in both *O. niloticus* (42.86% vs. 15.71%) and *C. gariepinus* (48.0% vs. 14.0%). These findings are consistent with Hassan et al.^20^ who identified *E. faecalis* among 59% of the examined *O. niloticus* along the Suez Canal, Egypt. A notably higher contamination rate was observed by Mendoza et al.^39^, who found 100% occurrence of *Enterococcus* spp. in *O. niloticus* from Minalin areas, Philippines due to water contamination from interconnected fish farms and the Maniango River. In contrast, lower prevalence rates were reported by El-Kader and Mousa-Balabel^40^ (2.7%) and Adamu et al.^41^ (4%), suggesting that environmental factors such as water quality, geographic location, and farming practices significantly influence bacterial occurrence^42^. The relatively high prevalence observed in our study may indicate a potential risk for fish health and food safety, especially if pathogenic or resistant strains are involved.

Enterococci are one of the most common bacterial pathogens resistant to antibiotics^34^. Our findings confirm that *E. faecium* isolates exhibit higher resistance rates than *E. faecalis*, consistent with previous reports^43^. Notably, 52% of *E. faecium* and 27% of *E. faecalis* were resistant to three antibiotics, while resistance to five antibiotics was observed in 22% and 10% of isolates, respectively. This differential resistance may reflect species-specific genomic adaptability, including the acquisition of transposons and plasmids via horizontal gene transfer^44^. Fish isolates showed the highest resistance to amoxicillin (88.89%), with substantial resistance also observed for fluoroquinolones, macrolides, and tetracyclines. These patterns align with prior studies^45,46^, though our data reveal even higher resistance rates in some cases, particularly among *E. faecalis* isolates. The variability in resistance profiles across geographic regions and fish species underscores the influence of local aquaculture practices and antibiotic usage. The antibiotic resistance in *E. faecalis* and *E. faecium* isolates from fish could be attributed to several factors. Importantly, our study identified a high prevalence of MDR and XDR enterococci: 70.83% of fish isolates were MDR and 29.17% were XDR. These rates exceed those reported by Mubarak et al.^14^, suggesting a worsening trend in resistance. We propose that the overuse of antibiotics as growth promoters, along with environmental contamination from poultry waste, and water from rivers that are polluted with urban sewage may contribute to the dissemination of resistance genes in aquatic ecosystems^39,47^. These findings emphasize the need for stricter regulations and surveillance to mitigate the spread of resistant enterococci from food sources to humans.

Vancomycin resistance poses a significant epidemiological threat, making it a major antibiotic resistance concern for enterococci^35^. VRE have seen a dramatic increase, globally rising from 0.05 to 99% in both humans and animals. VRE infections were responsible for 60–70% mortality globally^34^. In this study, VRE isolates were detected in 47.22% of fish samples and 75% of human isolates. These findings are consistent with previous studies, such as Lawpidet et al.^48^, who reported a 43.4% VRE prevalence in aquatic food, and Alemayehu and Hailemariam^49^, who reported a 74.8% VRE prevalence in human isolates from South Africa. Among the 34 VRE isolates recovered from fish samples in this study, 70.59% were identified as VREfm and 29.41% as VREfs. Previous studies have also reported VRE in fish and seafood. Igbinosa and Beshiru^45^ found vancomycin resistance in 37.3% of enterococci from ready-to-eat seafood. Additionally, Kukułowicz et al.^50^ reported a 21% VRE prevalence in fish samples from Tri-City, Poland, while Abdel-Raheem et al.^51^ identified VREfm in 37% of fish and crustacean samples from Port Said, Egypt.

In contrast to fish samples, human samples predominantly contain VREfm. Approximately 60% of VRE isolates from humans were *E. faecium*, and 40% were *E. faecalis*. This is consistent with other studies, such as Sivarajdy et al.^52^ and Shrestha et al.^53^, which reported higher prevalence of VREfm (81% and 22.40%, respectively) compared to VREfs (8% and 3.70%, respectively) in human samples.

As previously reported^34^, the most common genes of VREfs and VREfm in humans and animals are *vanA* and *vanB*. Our study revealed that the *vanA* gene was the most prevalent, detected in 83.33% of VREfs and 100% of VREfm isolates. The *vanB* gene was less common, occurring in 26.67% of VREfs and 15.79% of VREfm. The *vanC* gene was found in 13.33% of VREfs from both human and fish sources, but not in VREfm, consistent with Sivaradjy et al^52^ who reported *vanC* in 11% of VRE from Indian patients. Our study revealed that VRE isolates from fish were MDR, 38.24% and XDR, 61.76%, while human VRE isolates were MDR (60%) and XDR (40%). Additionally, 50% of VREfs and 47.37% of VREfm were resistant to quinupristin/dalfopristin. The emergence of MDR and XDR VRE, coupled with the presence of resistance determinants like *vanA* and *vanB*, poses a significant challenge for treatment, as these strains are often resistant to multiple classes of antibiotics^54^.

VRE harbor numerous genes encoding for variable virulence factors, enabling them to colonize, invade host tissues, causing disease in susceptible individuals and moreover, surviving under harsh conditions^55^. This study detected a diverse array of virulence genes, including *asa1*, *espE*, *ace*, *sprE*, *efaA*, *cylA*, *gelE*, and *hyl*, in both VREfs and VREfm isolates from human and fish sources. *ace* and *sprE* are associated with increased resistance to immune response and medication^10,56^. *efa* and *ace* were more prevalent in VREfs, suggesting higher virulence potential compared to VREfm^54^. *gelE* and *sprE* were the most frequent genes in VREfs (100%), consistent with previous findings^14,57^. *gelE* is believed to enhance survival in extraintestinal environments^58^. *asa1* was highly prevalent in VREfs (86.67%), aligning with previous reports^14,59^. *cylA* was the most frequent gene in VREfm (89.47%), followed by *gelE*, *sprE*, *asa1*, and *esp*. However, Sun et al.^60^ found *esp* in 76.2% and *hyl* in 66.7% of VREfm isolates, while *cylA* was found in only 10.7%. The presence of multiple virulence gene combinations in VREfs and VREfm highlights their potential for increased pathogenicity, as previously reported^10,54,61^.

Experimental infection of *O. niloticus* with VREfs and VREfm isolates confirmed their pathogenicity. The infected fish exhibited clinical signs and post-mortem lesions similar to those observed in naturally infected fish in our study and previous research^46,62^. The VREfs-infected group experienced a significantly higher mortality rate (100%) compared to the VREfm-infected group (60%). This difference can be attributed to the lower prevalence of virulence factors in VREfm compared to VREfs, as previously reported^28^.

MLST findings revealed the presence of both overlapped and host-specific STs among the examined *Enterococcus* isolates from humans and fish. The presence of *E. faecalis* ST21 in isolates sourced from both hosts suggests the wide host range of this ST beyond human. This was consistent with previous studies that reported the existence of ST21 in *E. faecalis* from humans and animals. In a study carried out by Zaheer et al.^63^, *E. faecalis* ST21 clone was recovered from different niches including beef processing, urban wastewater and human clinical sources. Another study reported the recovery of *E. faecalis* ST21 from treated wastewater revealing a close genetic relationship to clinical *E. faecalis* ST21 isolates from humans^64^. In contrast, the identification of ST273 only in fish, and ST283 and ST878 solely in human isolates, refers to host-specific strains that may be adapted to distinct ecological niches. This observation agreed with previous studies suggesting niche adaptation within *E. faecalis* populations^65,66^. A comparable pattern appears in *E. faecium*, where two distinct STs were identified from both hosts: ST218 in human and ST583 in fish isolates, reinforcing the idea of ST host specialization^65^. Taken together, the presence of both overlapped and host-specific STs reflects the complex ecology of *Enterococcus* species. These insights emphasize the need for continuous monitoring of microbial populations among human, animals, and environment using a one health strategy, for better controlling of emerging zoonotic threats. A key limitation is the geographical distance (~ 65 km) between the sampled fish farms and hospitals, which precludes direct analysis of transmission between these two reservoirs. Consequently, this study describes and compares the VRE isolates but cannot establish epidemiological links or confirm interspecies transmission. Future studies should sample farm workers and integrated aquaculture-hospitals communities to directly investigate transmission pathways.

## Conclusion

Circulation of VRE in fish populations indicates the role of the aquatic environment as a reservoir for VRE and the potential dissemination to humans. The high prevalence of MDR and XDR phenotypes among fish-derived enterococci raises serious concerns for food safety and public health. Our data suggests that unregulated antibiotic use may accelerate resistance gene dissemination across microbial communities. Moreover, different combinations of virulence genes were found in VREfs and VREfm, suggesting the potential for synergistic effects of multiple virulence factors and their combined impact on VRE pathogenesis and complicate treatment outcomes. Hence, to combat the spread of VRE in both fish and human populations, increased efforts in infection control and antibiotic stewardship, tailored to local resistance patterns, are crucial public health interventions. Our findings contribute to a growing body of evidence advocating for a One Health approach to antimicrobial resistance management.

## Supplementary Information

Below is the link to the electronic supplementary material.

Supplementary Material 1

## Data Availability

The datasets generated and analysed during the current study are available in this article and were deposited in GenBank (accession nos: PV931745-PV931793).
