# Supplementary material for: Dissemination of vancomycin-resistant Enterococcus faecalis and Enterococcus faecium between humans and fishes
Source: Sci Rep. 2026 Mar 7;16:8622. doi: 10.1038/s41598-026-36572-5 (PMC12976033; doi:10.1038/s41598-026-36572-5)
Supplement: Supplementary file 1 — Supplementary Material 1 [file 41598_2026_36572_MOESM1_ESM.doc]

**Table S1.** Target genes and oligonucleotide primers sequences used in the study

| **Primer use and target gene** | **Primer** | **Nucleotide sequence (5’→3’)** | **Amplicon size (bp)** | **Reference** |
| --- | --- | --- | --- | --- |
| **Enterococci identification** |  |  |  |  |
| *ddl* *E. faecalis* | E1-F  E2-R | ATCAAGTACAGTTAGTCT  ACGATTCAAAGCTAACTG | 941 | Dutka-Malen *et al.*^1^ |
| *ddl* *E. faecium* | F1-F  F2-R | GCAAGGCTTCTTCTTAGAGA  CATCGTGTAAGCTAACTTC | 535 |  |
| **Detection of *VAN* genes** |  |  |  |  |
| *VanA* | A1-F  A2- R | GGGAAAACGACAATTGC  GTACAATGCGGCCGTTA | 732 | Dutka-Malen *et al.* ^1^ |
| *VanB* | EB3-F  EB4-R | ACGGAATGGGAAGCCGA  TGCACCCGATTTCGTTC | 647 | Depardieu *et al.* ^2^ |
| *VanC* | EC5-F  EC8-R | ATGGATTGGTAYTKGTAT  TAGCGGGAGTGMCYMGTAA | 815 | Depardieu *et al.* ^2^ |
| **Detection of virulence genes** |  |  |  |  |
| *efa*A | F  R | GCCAATTGGGACAGACCCTC CGCCTTCTGTTCCTTCTTTGGC | 688 | Creti *et al.* ^3^ |
| *ace* | F  R | GGAATGACCGAGAACGATGGC GCTTGATGTTGGCCTGCTTCCG | 616 |  |
| *asa1* | ASA 11  ASA 12 | GCACGCTATTACGAACTATGA  TAAGAAAGAACATCACCACGA | 375 | Coque *et al*. ^4^ |
| *gelE* | GEL 11  GEL 12 | TATGACAATGCTTTTTGGGAT  AGATGCACCCGAAATAATATA | 213 | Willems *et al*.^5^ |
| *sprE* | F  R | GGTAAACCAACCAAGTGAATC  TTCTTCCGATTGACGCAAAA | 300 | Fridkin *et al*.^6^ |
| *cylA* | CYT I  CYT IIb | ACTCGGGGATTGATAGGC  GCTGCTAAAGCTGCGCTT | 688 | Galli *et al*. ^7^ |
| *esp* | ESP 14F  ESP 12R | AGATTTCATCTTTGATTCTTGG  AATTGATTCTTTAGCATCTGG | 510 | Su *et al*. ^8^ |
| *hyl* | HYL n1  HYL n2 | ACAGAAGAGCTGCAGGAAATG  GACTGACGTCCAAGTTTCCAA | 502 | Rice *et al*. ^9^ |
| **Housekeeping genes of**  ***E. faecalis* and *E.*** ***faecium*** |  |  |  |  |
| *gdh* | gdh-1 | GGCGCACTAAAAGATATGGT | 530 | Homan *et al*. ^10^  Ruiz-Garbajosa *et al*. ^11^  Ruiz-Garbajosa *et al*. ^11^ |
|  | gdh-2 | CCAAGATTGGGCAACTTCGTCCCA |  |  |
| *gyd* | gyd-1 | CAAACTGCTTAG CTCCAATGGC | 395 |  |
|  | gyd-2 | CATTTCGTTGTCATACCAAGC |  |  |
| *pstS* | pstS-1 | CGGA ACAGGACTTTCGC | 583 |  |
|  | pstS-2 | ATTTACATCACGTTCTACTTGC |  |  |
| **Housekeeping genes of**  ***E. faecalis***  *gki* | gki-1 | GATTTTGTGGGAATTGGTATGG | 438 |  |
|  | gki-2 | ACCATTAAAGCAAAATG ATCGC |  |  |
| *aroE* | aroE-1 | TGGAAAACTTTACGGAGACAGC | 459 |  |
|  | aroE-2 | GTCCTG TCCATTGTTCAAAAGC |  |  |
| *xpt* | xpt-1 | AAAATGATGGCCGTGTATTAGG | 456 |  |
|  | xpt-2 | AACGTCACCGTTCCTTCACTTA |  |  |
| *yqiL* | yqiL-1 | CAGCTTAAGTCAAG TAAGTGCCG | 436 |  |
|  | yqiL-2 | GAATATCCCTTCTGCTTGTGCT |  |  |
| **Housekeeping genes of**  ***E.*** ***faecium*** |  |  |  | Homan *et al*.^10^ |
| *purK* | purK1 | GCA GAT TGG CAC ATT GAA AGT | 492 |  |
|  | purK2 | TAC ATA AAT CCC CCT GTT TY |  |  |
| *adk* | adk1 | TAT GAA CCT CAT TTT AAT GGG | 437 |  |
|  | adk2 | GTT GAC TGC CAA ACG ATT TT |  |  |
| *atpA* | atpA1 | CGG TTC ATA CGG AAT GGC ACA | 556 |  |
|  | atpA2 | AAG TTC ACG ATA AGC CAC GG |  |  |
| *ddl* | ddl1 | GAG ACA TTG AAT ATG CCT TAT G | 465 |  |
|  | ddl2 | AAA AAG AAA TCG CAC CG |  |  |

1. Dutka-Malen, S., Evers, S. & Courvalin, P. Detection of glycopeptide resistance genotypes and identification to the species level of clinically relevant enterococci by PCR. *J. Clin. Microbiol.* **33**, 1434 (1995) doi: 10.1128/jcm.33.5.1434-1434.1995. Erratum for: *J Clin Microbiol.* 1995;**33**:24-7. doi: 10.1128/jcm.33.1.24-27.

2. Depardieu, F., Perichon, B. & Courvalin, P. Detection of the van alphabet and identification of enterococci and staphylococci at the species level by multiplex PCR. *J. Clin. Microbiol.* **42**, 5857-5860 (2004).

3. Creti, R. et al. Survey for virulence determinants among *Enterococcus faecalis* isolated from different sources. *J. Med. Microbiol.* **53**, 13-20 (2004).

4. Coque, T.M., Patterson, J.E., Steckelberg, J.M. & Murray, B.E. Incidence of hemolysin, gelatinase, and aggregation substance among enterococci isolated from patients with endocarditis and other infections and from feces of hospitalized and community-based persons. *J. Infect. Dis.* **171**, 1223-1229 (1995).

5. Willems, R. et al. Prevalence of esp, Encoding the Enterococcal Surface Protein, in Enterococcus faecalis and *Enterococcus faecium* isolates from hospital patients, poultry, and pigs in Denmark. *J. Clin. Microbiol.* 4396 (2002).

6. Fridkin, S.K. et al. The effect of vancomycin and third-generation cephalosporins on prevalence of vancomycin-resistant enterococci in 126 U.S. adult intensive care units. *Ann. Intern. Med.* **135**, 175-183 (2001).

7. Galli, D., Lottspeich, F. & Wirth, R. Sequence analysis of Enterococcus faecalis aggregation substance encoded by the sex pheromone plasmid pAD1. *Mol. Microbiol.* **4**, 895-904 (1990).

8. Su, Y. et al. Nucleotide sequence of the gelatinase gene (gelE) from *Enterococcus faecalis subsp. liquefaciens*. *Infection and immunity* **59**, 415-420 (1991).

9. Rice, L.B. et al. A potential virulence gene, hyl Efm, predominates in *Enterococcus* *faecium* of clinical origin. *J. Infect. Dis.* **187**, 508-512 (2003).

10. Homan, W.L. et al. Multilocus sequence typing scheme for *Enterococcus faecium*. *J Clin Microbiol* **40**, 1963-1971 (2002).

11. Ruiz-Garbajosa, P. et al. Multilocus sequence typing scheme for *Enterococcus faecalis* reveals hospital-adapted genetic complexes in a background of high rates of recombination. *J Clin Microbiol* **44**, 2220-2228 (2006).

**Table S2**. Antimicrobial resistance profile of *E. faecalis* and *E. faecium* isolates from humans and fish

| **Antibiotics** | **No. of resistant isolates (%)** | | | | | | | | | |
| --- | --- | --- | --- | --- | --- | --- | --- | --- | --- | --- |
|  | ***E. faecalis* (n= 64)** | | | | | ***E. faecium* (n= 28)** | | | | |
|  | ***O. niloticus* (n= 30)** | ***C.gariepinus***  **(n= 24)** | **Human**  **(n= 10)** | ***p-*value** | **Total**  **(n= 64)** | ***O. niloticus* (n= 11)** | ***C. gariepinus* (n= 7)** | **Human**  **(n= 10)** | ***p-*value** | **Total (n= 28)** |
| Ampicillin | 17 (56.67) | 7 (29.17) | 6 (60) | 0.088 | 40 (62.5) | 5 (45.45) | 4 (57.14) | 6 (60) | 0.781 | 15 (53.57) |
| Amoxicillin | 27 (90) | 22 (91.67) | 5 (50) | 0.005** | 54 (84.4) | 8 (72.73) | 7 (100) | 4 (40) | 0.03* | 19 (67.86) |
| Ciprofloxacin | 26 (86.67) | 11 (45.83) | 8 (80) | 0.004** | 45 (70.3) | 7 (63.63) | 7 (100) | 8 (80) | 0.185 | 22 (78.57) |
| Levofloxacin | 20 (66.67) | 11 (45.83) | 6 (60) | 0.302 | 37 (57.8) | 7 (63.63) | 7 (100) | 8 (80) | 0.185 | 22 (78.57) |
| Norofloxacin | 23 (76.67) | 10 (41.67) | 6 (60) | 0.032* | 39 (60.9) | 10 (90.91) | 7 (100) | 7 (70) | 0.18 | 24 (85.71) |
| Ofloxacin | 26 (86.67) | 11 (45.83) | 6 (60) | 0.006** | 43 (67.2) | 7 (63.63) | 7 (100) | 8 (80) | 0.185 | 22 (78.57) |
| Linezolid | 0 | 0 | 0 | NA | 0 | 0 | 0 | 0 | NA | 0 |
| Tigecycline | 19 (63.33) | 9 (37.5) | 7 (70) | 0.095 | 35 (54.7) | 5 (45.45) | 7 (100) | 4 (40) | 0.029* | 16 (57.14) |
| Gentamicin | 17 (56.67) | 6 (25.00) | 7 (70) | 0.019* | 30 (46.9) | 5 (45.45) | 6 (85.71) | 8 (80) | 0.121 | 19 (67.86) |
| Streptomycin | 14 (46.67) | 8 (33.33) | 5 (50) | 0.53 | 27 (42.2) | 4 (36.36) | 5 (71.43) | 6 (60) | 0.305 | 15 (53.57) |
| Imipenem | 10 (33.33) | 8 (33.33) | 4 (40) | 0.92 | 22 (34.4) | 5 (45.45) | 3 (42.86) | 6 (60) | 0.728 | 14 (50) |
| Meropenem | 7 (23.33) | 6 (25.00) | 3 (30) | 0.915 | 16 (25) | 4 (36.36) | 3 (42.86) | 3 (30) | 0.861 | 10 (35.71) |
| Erythromycin | 26 (86.67) | 9 (37.5) | 8 (80) | <0.0001*** | 43 (67.2) | 7 (63.63) | 7 (100) | 7 (70) | 0.114 | 21 (75) |
| Azithromycin | 21 (70.00) | 8 (33.33) | 6 (60) | 0.025* | 35 (54.7) | 4 (36.36) | 6 (85.71) | 7 (70) | 0.085 | 17 (60.71) |
| Clindamycin | 26 (86.67) | 10 (41.67) | 7 (70) | 0.002** | 43 (67.2) | 4 (36.36) | 7 (100) | 8 (80) | 0.011* | 19 (67.86) |
| Vancomycin | 17 (56.67) | 7 (29.17) | 6 (60) | 0.088 | 30 (46.9) | 4 (36.36) | 6 (85.71) | 9 (90) | 0.016* | 19 (67.86) |
| Teicoplanin | 15 (50) | 4 (16.67) | 3 (30) | 0.036* | 22 (34.4) | 4 (36.36) | 4 (57.14) | 7 (70) | 0.297 | 15 (53.57) |
| Chloramphenicol | 22 (73.33) | 9 (37.5) | 7 (70) | 0.022* | 38 (59.4) | 6 (54.55) | 7 (100) | 8 (80) | 0.085 | 21 (75) |
| Nitrofurantoin | 17 (56.67) | 8 (33.33) | 6 (60) | 0.17 | 31 (48.4) | 6 (54.55) | 5 (71.43) | 8 (80) | 0.447 | 19 (67.86) |
| Tetracycline | 19 (63.33) | 11 (45.83) | 8 (80) | 0.151 | 38 (59.4) | 5 (45.45) | 4 (57.14) | 7 (70) | 0.525 | 16 (57.14) |
| Daptomycin | 17 (56.67) | 6 (25) | 6 (60) | 0.04* | 29 (45.3) | 4 (36.36) | 6 (85.71) | 5 (50) | 0.118 | 15 (53.57) |
| Quinupristin-dalfopristin | 8 (26.67) | 4 (16.67) | 4 (40) | 0.344 | 16 (25) | 3 (27.27) | 3 (42.86) | 3 (30) | 0.775 | 9 (32.14) |

NA: non-applicable. **p* <0.05, ***p* <0.01, ****p* < 0.001.

**Table S3.** The identified sequence types and allelic designation for housekeeping genes in the MLST scheme among Vancomycin-resistant *E. faecalis* and *E. faecium* strains from humans and fishes sample

| **STs** | **Strains** | **Source** | **Alleles** | **Accession nos.** |
| --- | --- | --- | --- | --- |
| ST21 | VREfs36  VREfs49  VREfs50  VREfs1 | Human urine  *C. gariepinus* spleen  *O. niloticus* ascitic fluid  *O. niloticus* kidney | *gdh* Allele 1  *gyd* Allele 7  *pstS* Allele 9  *gki* Allele 1  *aroE* Allele 1  *xpt* Allele 1  *yqiL* Allele 1 | PV931745  PV931749  PV931753  PV931757  PV931761  PV931768  PV931772 |
| ST273 | VREfs16 | *C. gariepinus* spleen | *gdh* Allele 4  *gyd* Allele 8  *pstS* Allele 12  *gki* Allele 13  *aroE* Allele 40  *xpt* Allele 46  *yqiL* Allele 22 | PV931754  PV931762  PV931746  PV931773  PV931758  PV931769  PV931750 |
| ST283 | VREfs38 | Human urine | *gdh* Allele 1  *gyd* Allele 7  *pstS* Allele 11  *gki* Allele 11  *aroE* Allele 3  *xpt* Allele 4  *yqiL* Allele 2 | PV931747  PV931755  PV931763  PV931759  PV931770  PV931751  PV931774 |
| ST878 | VREfs35 | Human urine | *gdh* Allele 34  *gyd* Allele 2  *pstS* Allele 17  *gki* Allele 37  *aroE* Allele 29  *xpt* Allele 23  *yqiL* Allele 17 | PV931771  PV931760  PV931775  PV931752  PV931756  PV931764  PV931748 |
| ST218 | VREfm42 | Human blood | *gdh* Allele 8  *gyd* Allele 6  *pstS* Allele 10  *purK* Allele 23  *adk* Allele 11  *atpA* Allele 13  *ddl* Allele 8 | PV931779  PV931782  PV931785  PV931765  PV931776  PV931788  PV931791 |
| ST583 | VREfm9 | *O. niloticus* spleen | *gdh* Allele 14  *gyd* Allele 8  *pstS* Allele 26  *purK* Allele 22  *adk* Allele 6  *atpA* Allele 5  *ddl* Allele 8 | PV931786  PV931789  PV931783  PV931780  PV931777  PV931792  PV931766 |
| *Nearest ST: ST695, ST834, ST2262, ST1550 | VREfm6 | *C. gariepinus* liver | *gdh* Allele 8  *gyd* Allele 10  *pstS* Allele 19  *purK* Allele 8  *adk* Allele 6  *atpA* Allele 11*  *ddl* Allele 8 | PV931778  PV931790  PV931784  PV931787  PV931767  PV931793  PV931781 |

ST: sequence type, VREfs: Vancomycin-resistant *E. faecalis*, VREfm: Vancomycin-resistant *E. faecium*.* The identity of *atpA* is 99.82%.

**
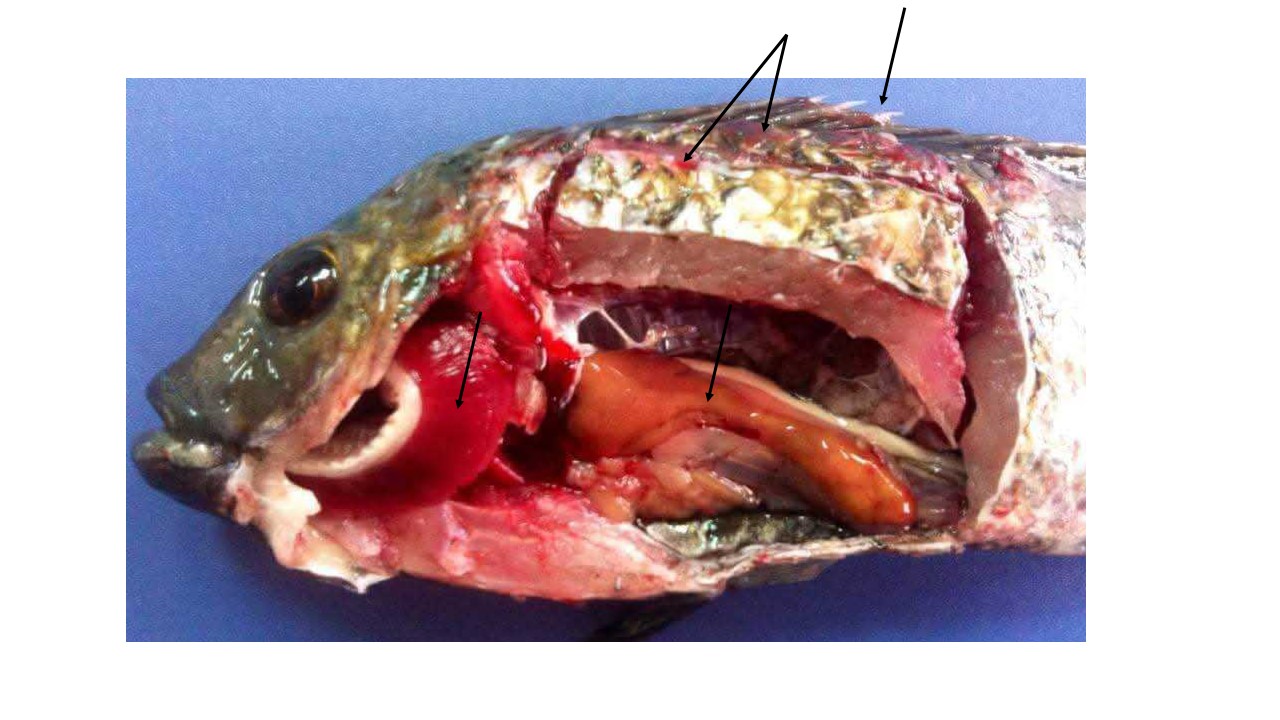
**

**Figure S1.** *Oreochromis niloticus* experimentally infected with *E. faecalis* showing erosions of dorsal fin, congested gills, hemorrhage on the body, fins, and liver.
